# Wild and Cultivated *Centaurea raphanina* subsp. *mixta*: A Valuable Source of Bioactive Compounds

**DOI:** 10.3390/antiox9040314

**Published:** 2020-04-15

**Authors:** Spyridon A. Petropoulos, Ângela Fernandes, Maria Ines Dias, Carla Pereira, Ricardo Calhelha, Francesco Di Gioia, Nikolaos Tzortzakis, Marija Ivanov, Marina Sokovic, Lillian Barros, Isabel C. F. R. Ferreira

**Affiliations:** 1 Department of Agriculture Crop Production and Rural Environment, University of Thessaly, Fytokou Street, 38446 N. Ionia, Greece; 2 Centro de Investigação de Montanha (CIMO), Instituto Politécnico de Bragança, Campus de Santa Apolónia, 5300-253 Bragança, Portugal; afeitor@ipb.pt (Â.F.); maria.ines@ipb.pt (M.I.D.); carlap@ipb.pt (C.P.); calhelha@ipb.pt (R.C.); lillian@ipb.pt (L.B.); 3 Department of Plant Science, The Pennsylvania State University, University Park, Pennsylvania, PA 16802, USA; fxd92@psu.edu; 4 Department of Agricultural Sciences, Biotechnology and Food Science, Cyprus University of Technology, Lemesos 3603, Cyprus; nikolaos.tzortzakis@cut.ac.cy; 5 Institute for Biological Research “Siniša Stanković”-National Institute of Republic of Serbia, University of Belgrade, Bulevar despota Stefana 142, 11060 Belgrade, Serbia; marija.smiljkovic@ibiss.bg.ac.rs (M.I.); mris@ibiss.bg.ac.rs (M.S.)

**Keywords:** antimicrobial activities, antioxidant activity, cytotoxic effects, organic acids, phenolic compounds

## Abstract

*Centaurea raphanina* subsp. *mixta* (DC.) Runemark is a wild edible species endemic to Greece. This study evaluated the chemical composition and bioactive properties of wild and cultivated *C. raphanina* subsp. *mixta* plants. Wild plants had higher nutritional value than cultivated ones, whereas cultivated plants contained more tocopherols. Glucose and sucrose were higher in cultivated plants and trehalose in wild ones. Oxalic and total organic acids were detected in higher amounts in cultivated samples. The main fatty acids were α-linolenic, linoleic and palmitic acid, while wild plants were richer in polyunsaturated fatty acids. Two pinocembrin derivatives were the main phenolic compounds being detected in higher amounts in wild plants. Regarding the antioxidant activity, wild and cultivated plants were more effective in the oxidative haemolysis (OxHLIA) and thiobarbituric acid reactive substances (TBARS) assays, respectively. Moreover, both extracts showed moderate cytotoxicity in non-tumor cell lines (PLP2), while cultivated plants were more effective against cervical carcinoma (HeLa), breast carcinoma (MCF-7) and non-small lung cancer (NCI-H460) cell lines. Finally, wild plants showed higher antimicrobial activity than cultivated plants against specific pathogens. In conclusion, the cultivation of *C.* *raphanina* subsp. *mixta* showed promising results in terms of tocopherols content and antiproliferative effects, however further research is needed to decrease oxalic acid content.

## 1. Introduction

Wild edible species or wild greens represent a rich patrimony of the Mediterranean basin with several uses in local and traditional cuisine, as well as in traditional and folk medicine [1,2,3,4]. Several research studies highlighted the important beneficial health effects of various wild species that constitute common ingredients of the so called Mediterranean diet [5,6]. These effects are mostly associated with their high content in phytochemicals such as flavanones [7], sterols [6], phenolic acids [8,9,10], sesquiterpene lactones [11,12,13], omega-3 fatty acids [14,15] and other secondary metabolites with bioactive and antioxidant properties. The increasing demand for functional foods has put wild edible greens in the center of attention and many recent studies have highlighted the potential of commercial exploitation of wild plants that may diversify modern diets and increase throughout the year the availability of such products [9,16,17,18,19,20]. However, domestication of wild species needs several aspects to be considered since various reports highlighted significant changes of bioactive compounds content in domesticated species compared to their wild counterparts, while scarce literature reports exist regarding their agronomic requirements [17,21].

**Figure 1:**
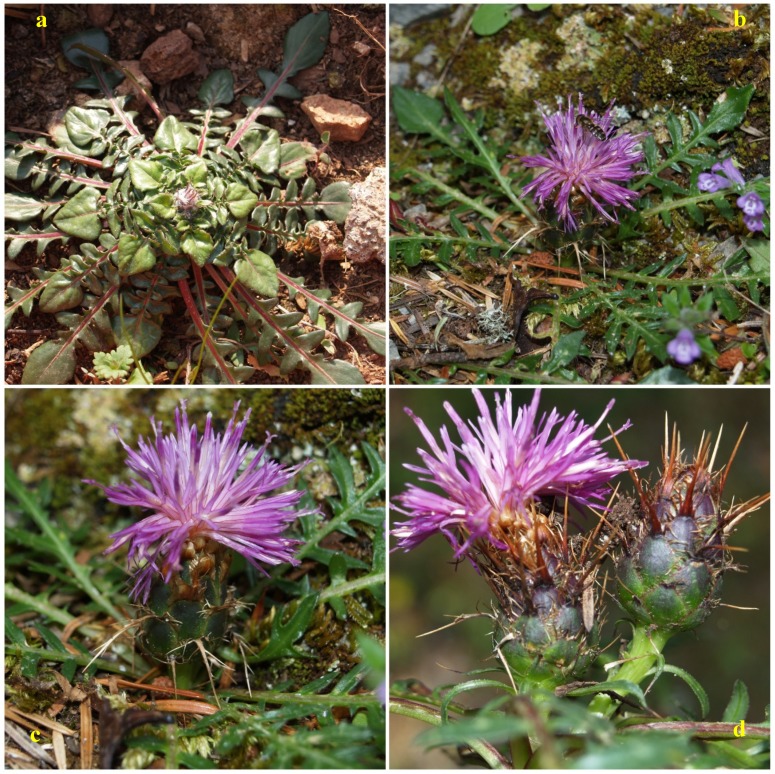
Photographs of wild *Centaurea raphanina* spp. *mixta* before (**a**) and during anthesis (**b**–**d**). Photo credits: Spyridon A. Petropoulos.

The genus *Centaurea* includes a large number of species (more than 500) belonging to the Asteraceae family, which are commonly found in the broader region of the Mediterranean basin and Western Asia [22]. The genus consists of very diverse species with different growth cycle (annual, biennial or perennial plants) and growth habits (edible greens, herbs and bushes), while some of them are traditionally used as edible greens or as medicinal plants due to their bioactive properties [16,23,24,25,26]. Among these species, 134 are endemic as for example *Centaurea raphanina* spp. *mixta* (commonly known in Greece as agginaráki or alivárvaron) [27]. It is a perennial plant widely distributed in Greece with a long and thick taproot which allows for growing under arduous conditions, such as high altitudes, rocky slopes and low temperatures. It is highly esteemed for its edible tender leaves which form a rosette, while its small flowers resemble the flower of globe artichoke, hence its common Greek name “agginaráki” which means a small artichoke (Figure 1). The limited existing reports for the species refer to its antifungal properties, where according to Panagouleas et al. [27] leaf extracts showed strong in vitro activity against various fungi due to the presence of a sesquiterpene lactone, namely cnicin. In a recent study, Mikropoulou et al. [7] reported the phytochemical composition of decoctions from the species and detected five flavanones as the major compounds, namely phlorin, syringin, pinocembrin, pinocembroside, and pinocembrin-7-*O*-neohesperidoside. The obtained decoctions were also reported to show toxicity against A5 cancer metastatic cancer cells, whereas the evaluation of antioxidant activity through the DPPH assay showed low free radical scavenging activity [7]. According to Sokovic et al. [12], the biological activities of *Centaurea* species is mostly associated with the presence of flavonoids as well as sesquiterpene lactones (germacranolides, eudesmanolides, elemanolides, and guaianolides). 

Despite the increasing number of reports for the bioactivities and health benefits of wild edible plants [27,28,29,30], more studies are needed to reveal the impact of cultivation practices on tended plants before suggesting their introduction to commercial cultivation. The need to cultivate wild edible species arises from the increasing demand of safe and high-quality product of local restaurants and consumers and the obligation to avoid irrational harvesting of these species and preserve their natural habitat [31,32]. Most of the existing reports refer to phytochemical analyses and determination of bioactive properties of wild edible species collected from their natural habitats, without many comparative studies between wild and cultivated species. For example, according to Çekiç and Özgen [33] who compared wild accessions of raspberries (*Rubus idaeus* L.) with commercial cultivars, a high variation in antioxidant capacity and phytonutrients content among the wild accessions was observed while some of the tested accessions showed better results than the commercial cultivars. Similarly, Isbilir and Sagiroglu [34] reported a higher antiradical and antioxidant activity and a higher total phenolics content for extracts obtained from wild sheep sorrel (*Rumex acetosella* L.) plants than extracts from cultivated ones. In the study of Disciglio et al. [35], samples from *Cichorium intybus* L., *Borago officinalis* L. and *Diplotaxis tenuifolia* (L.) DC collected from wild and cultivated plants were compared in terms of dry matter, proteins, nitrates and polyphenols content, as well as regarding their antioxidant activity. The reported results showed that wild plants had a higher quality than cultivated counterparts, since they contained higher amounts of the tested nutrients and polyphenols and a lower amounts of anti-nutritional factors such as nitrates [35]. Papafilippaki and Nikolaidis [36] carried out a comparative study between two wild (coastal and mountainous) and one cultivated population of *Cichorium spinosum* L. under the same growing conditions and reported a great variation in biomass production, mineral composition, arbuscular mycorrhizal colonization rate and biomass production, as well as a significant phenotypic variation among the studied genotypes. In the same context, Alu’datt et al. [37] compared nutritional value, phenolic compounds content and antioxidant activity of wild, cultivated (soil and soilless cultivation) and obtained from the market purslane (*Portulaca oleracea* L.) leaves and suggested that soilless cultivation or the use of growth substrates such as tuff, peat moss, perlite and zeolitic tuff may increase nutritional quality and bioactive compounds content of the final product. 

So far, scarce literature reports regarding the proximate and chemical composition of *C. raphanina* spp. *mixta* exist, while all the existing studies refer to samples collected from the wild. Therefore, taking into consideration the increasing needs of consumers and the market requests for alternative healthy and functional food products, as well as the lack of available information in the scientific literature, the present study aimed to evaluate: (i) nutritional value, bioactive properties and chemical composition differences between wild and cultivated plants of *C. raphanina* spp. *mixta*, and (ii) the potential of suggesting the species as a novel and alternative food crop with high added value and functional properties. Finally, in contrast to most of the existing studies where different genotypes are tested under wild and domesticated conditions, the novel aspect of this study is that the studied genotype was the same for both wild and cultivated plants which allows to make safe conclusions from their direct comparison. 

## 2. Materials and Methods 

### 2.1. Plant Material and Growing Conditions

**Figure 2:**
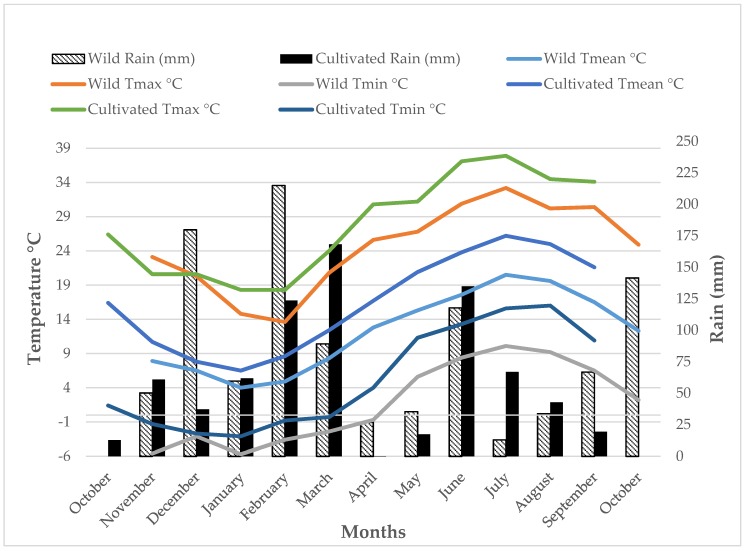
Growth conditions of wild and cultivated plants throughout the growing period. For cultivated plants, the presented temperature (mean, maximum and minimum) and rain values refer to the October 2017–September 2018 period (Velestino site), while for wild plants the presented temperature (mean, maximum and minimum) and rain values refer to the growing period before harvest (September 2016–October 2017; Mainalo site).

Wild plants of *Centaurea raphanina* spp. *mixta* were collected in October 2017 at Mainalo mountain in the Arcadia prefecture, Greece (37.62 N, 22.10 E, altitude: 1194 m above sea level). Fully expanded, tender and healthy leaves were taken from 50 different plants in a range of 500 m in order to capture population heterogeneity. After harvest, leaves were put immediately in food bags and stored at 4 °C until transfer to the laboratory of Vegetable Production, University of Thessaly, Greece. Moreover, whole plants (30 randomly selected and uniform in size plants) were removed with soil from the same location trying not to disturb as much as possible its taproot. After removal, plants were transplanted in 2 L pots which were filled with peat substrate (Klassman-Deilmann KTS2) and transferred to the experimental farm of the University in Velestino, Greece (39.39 N, 22.75 E; altitude: 81 m above sea level). Considering the perrenial nature of the species and the long taproot of the species, after the first establishment plants were transferred to 10 L pots filled with the same peat subtrate and perlite in a ratio of 2:1 (*v/v*) and left outdoors throughout the growing season and until harvest. Growth conditions of wild and cultivated plants are presented in Figure 2. Irrigation was carried out regularly and according to environmental conditions, while fertilization was carried out monthly with fertigation solution containing 200 mg/L of a N-P-K water soluble complex fertilizer Atlas 20-20-20 + TE containing 20% N (5.8% NO_3_^−^, 4% NH_4_^+^ and 10.2% urea), 20% P_2_O_5_, 20% K_2_O, plus 0.0033% Cu, 0.039% Fe, 0.0163% Mn, 0.0033% Zn, 0.0016 Mo, 0.0163% B (Gavriel S.A., Greece). No pesticides were used during cultivation. Harvest of cultivated plants took place the next growing season on October 2018 by collecting fully expanded, tender and healthy leaves of marketable size and based on the methodology described by the authors [10]. Fresh leaves were combined in batch samples and put at −80 °C until lyophilization, ground to powder by using a pestle and mortar, and finally put in air-sealed plastic food bags and stored again at −80 °C until the chemical analyses were performed.

### 2.2. Chemical Analyses Assays

#### 2.2.1. Nutritional Value and Energetic Value

Moisture, fat, protein, ash, carbohydrates and energy were estimated according to the Association of Official Analytical Chemists (AOAC) procedures [38]. Total carbohydrates were calculated by difference: total carbohydrates (g/100 g fresh weight (fw)) = 100 − (g moisture + g fat + g ash + g proteins) and total energy was calculated according to the following equation: energy (kcal/100 g fw) = 4 × (g proteins + g carbohydrates) + 9 × (g fat) [38].

#### 2.2.2. Free Sugars

Free sugars were determined following a procedure previously optimized and described by Guimarães et al. [39], using high performance liquid chromatography (HPLC) coupled to a refraction index detector (RI). Sugars standards were used for identification by chromatographic comparison, using the internal standard (melezitose) method. The results were presented as g/100 g of fw.

#### 2.2.3. Organic Acids

Organic acids were analysed using UFLC (Ultra-Fast Liquid Chromatography; Shimadzu 20A series, Kyoto, Japan) and a photo-diode array detector, as previously optimized and described by Pereira et al. [40]. The results were presented as mg/100 g of fw.

#### 2.2.4. Tocopherols

Tocopherols determination was based on the method of Guimarães et al. [39], by HPLC coupled to a fluorescence detector (FP-2020; Jasco, Easton, MD, USA) programmed for excitation at 290 nm and emission at 330 nm. Quantification was based on the fluorescence signal response of each standard, using the IS (tocol, 50 mg/mL) method and calibration curves obtained from commercial standards of each compound. The results were presented as mg/100 g of fw.

#### 2.2.5. Fatty Acids

Fatty acids were analysed using a GC-FID (gas-liquid chromatography with flame ionization detection, GC-FID; DANI1000, Contone, Switzerland) equipment with a capillary column as described previously [39]. The results were presented as a relative percentage of each fatty acid.

#### 2.2.6. Phenolic Compounds

The evaluation the phenolic compounds and bioactive properties was performed in hydroethanolic extracts of the samples which were prepared by stirring 1 g of the dried plant material with 30 mL of ethanol-water (80:20, *v/v*, at 25 °C) for 60 min [41]. The obtained extracts were filtered through Whatman paper No. 4 filters [41]. The residue was then re-extracted with the addition of 30 mL of the hydroethanolic solution and filtered as above. The extracts obtained from the above procedure were combined and then evaporated under reduced pressure (Büchi R-210, rotary evaporator, Flawil, Switzerland) until ethanol was completely removed. After evaporation, the residues (aqueous phase) were put to freezing until lyophilisation was performed (FeeeZone 4.5, Labconco, Kansas City, MO, USA).

Phenolic compounds were determined in the hydroethanolic extracts prepared (see description above), after they were re-dissolved in ethanol/water (80:20, *v/v*) to a final concentration of 10 mg/mL and filtered through a 0.22 μm disposable filter disk [41]. The extracts were analyzed using a Dionex Ultimate 3000 (Dionex Ultimate 3000 UPLC and Linear Ion Trap LTQ XL, Thermo Scientific, San Jose, CA, USA) ultra-performance liquid chromatography system equipped with a diode array detector coupled to an electrospray ionization mass spectrometry detector [41]. The acquisition of data acquisition and their processing was carried out with the Xcalibur^®^ data system (Thermo Scientific, San Jose, CA, USA). The identification of individual phenolic compounds was performed by comparing retention times, UV–visible spectra and MS fragmentation pattern of detected compounds with those of authentic standards (Extrasynthése S.A., Genay, France). Moreover, for the identification of phenolic compounds the data available from the literature were also used. The quantification of the detected phenolic compounds was based on calibration curves of authentic standards. The results were presented as mg/100 g fw.

### 2.3. Antioxidant Activity

The antioxidant activity was evaluated by applying two cell-based assays in the already prepared hydroethanolic extracts (see Section 2.2): the oxidative haemolysis (OxHLIA) and the thiobarbituric acid reactive substances (TBARS) formation inhibition assays previously described by Lockowandt et al. [42], using the above prepared hydroethanolic extracts. The used positive control was Trolox. 

#### 2.3.1. OxHLIA Assay

The antihaemolytic activity was evaluated by the oxidative haemolysis inhibition assay (OxHLIA) [42]. The results were presented as IC_50_ values, which is the extract concentration (µg/mL) required to inhibit oxidative haemolysis of 50% of the erythrocytes for *Δ*t of 60 min.

#### 2.3.2. TBARS Assay

For the TBARS assay, the pig (*Sus scrofa*) brain tissues were dissected and homogenized with a Tris-HCl buffer (20 mM, pH 7.4) to obtain a homogenate (1:2; *w/v*) of brain tissue which was then centrifuged for 10 min at 3000× *g* [42]. The extract samples (0.2 mL) were incubated with the porcine brain supernatant (1:2, *w/v*; 0.1 mL) at 37 °C for 1 h after the addition of FeSO_4_ (10 µM; 0.1 mL) and ascorbic acid (0.1 mM; 0.1 mL). Then, tri-chloroacetic (28% *w/v*, 0.5 mL) and thiobarbituric (TBA, 2%, *w/v*, 0.38 mL) acids were added and the mixture was heated at 80 °C for 20 min and centrifuged for 5 min at 3000× *g* [42]. The results were presented as EC_50_ values, which is the extract concentration (μg/mL) that provides 50% of antioxidant activity.

### 2.4. Hepatotoxicity and Cytotoxicity Assays

Hepatotoxicity was evaluated using the Sulforhodamine B (Sigma-Aldrich, St Louis, MO, USA) assay [43]. Briefly, primary cell culture (PLP2) were prepared from porcine liver and tested with different concentrations of the above described hydroethanolic extracts (see Section 2.3). The anti-proliferative capacity of the extracts was also evaluated by using four human tumor cell lines (acquired from Leibniz-Institut DSMZ, Braunschweig, Germany), namely HeLa (cervical carcinoma), HepG2 (hepatocellular carcinoma), MCF-7 (breast adenocarcinoma) and NCI-H460 (non-small cell lung cancer) [43]. In both hepatotoxicity and cytotoxicity assays, ellipticine (Sigma-Aldrich, St Louis, MO, USA) was used as the positive control and the results were presented as GI_50_ values (μg/mL), which corresponds to the extract concentration that inhibits cell growth by 50%.

### 2.5. Antimicrobial Properties

In order to determine potential antimicrobial activity of the samples, the Gram-positive bacteria: *Staphylococcus aureus* (American Type Culture Collection, Manassas, VA, USA, ATCC 6538), *Bacillus cereus* (food isolate), *Listeria monocytogenes* (National Collection of Type Cultures, London, UK, NCTC 7973), as well as the following Gram-negative bacteria: *Escherichia coli* (ATCC 25922), *Salmonella typhimurium* (ATCC 13311) and *Enterobacter cloacae* (ATCC 35030) were used. For antifungal assays, six micromycetes were used: *Aspergillus fumigatus* (ATCC 9197), *Aspergillus niger* (ATCC 6275), *Aspergillus versicolor* (ATCC 11730), *Penicillium funiculosum* (ATCC 36839), *Trichoderma viride* (IAM Culture Collection, Center for Cellular and Molecular Research, Institute of Molecular and Cellular Biosciences, The University of Tokyo, Tokyo, Japan (collection transferred to JCM), IAM 5061) and *Penicillium verrucosum* var. *cyclopium* (food isolate). For the antimicrobial properties of the extracts, the microdilution method was used [44]. The results were presented as the concentrations that resulted in complete inhibition of the bacterial growth (MIC, minimal inhibition concentration), through the colorimetric microbial viability assay, as well as MBC and MFC values (minimal bactericidal concentration and minimal fungicidal concentration, respectively). The used positive controls were streptomycin, ampicillin, ketoconazole and bifonazole (Sigma-Aldrich, St. Louis, MO, USA), whereas the negative control was 5% DMSO.

### 2.6. Statistical Analysis

The experimental design was a completely randomized design (CRD) in the case of cultivated plants with 30 replications (*n* = 30). For chemical composition and bioactivity assays three samples were analyzed for wild and cultivated plants and all the assays were carried out in triplicate. Statistical analysis was performed with the use of Statgraphics 5.1.plus (Statpoint Technologies, Inc., VA, USA). Data were evaluated by one-way ANOVA for the main effect, while *t*-Student test (*p* = 0.05) was used for the comparison of means. 

## 3. Results and Discussion

### 3.1. Nutritional Value and Chemical Composition

#### 3.1.1. Proximate Analysis and Energetic Value

The nutritional and the energetic value of the leaves of wild and cultivated *Centaurea raphanina* spp. *mixta* plants are presented in Table 1. Moisture content was higher in cultivated plants (93.3 g/100 g fw compared to 84.8 g/100 g fw of wild plants) due to regular irrigation that cultivated plants received during the growing period, whereas fat, protein, ash and carbohydrates content (0.42, 2.82, 2.17 and 9.7 g/100 g fw) and energetic value (54.1 kcal/100 g fw) were higher in leaves harvested from wild plants. Similar results of moisture content have been reported for other wild leafy vegetables by Boari et al. [45] who studied the antioxidant profile of four wild leafy Asteraceae (*Helminthotheca echioides* L., *Sonchus oleraceus* L., *Taraxacum officinale* Weber, *Urospermum picroides* (L.) Schmidt) and other wild edible species collected in Southern Italy, and by Afolayan and Jimoh [46] who studied the nutritional value of *Sonchus asper* (L.) Hill and *Chenopodium album* L. among other wild leafy species. The higher moisture content of the cultivated plants may also be due to the higher availability of nitrogen and especially nitrate nitrogen periodically applied through fertigation, while wild plants were not fertilized. These results are in agreement with the negative relationship observed in previous studies between available nitrate-nitrogen and dry matter in lettuce and other crops [47,48]. The macronutrients content is also within the same range of other reports related with wild edible greens [10,49], which highlights the overall low contribution of these type of plants to daily dietary intake of macronutrients, especially when considering the low amounts usually consumed on a daily basis and the limited availability of wild plants throughout the year. The differences observed in our study between wild and cultivated plants could be ascribed to a concentration effect due to the lower moisture content of wild leaves although these differences are minimized when results are calculated on a dry weight basis. According to Zushi and Matsuzoe [50], this pattern can be observed in tomato fruit grown under salinity stress, while Giuffrida et al. [51], Di Gioia et al. [52], and Zaghdoud et al. [53] reported a similar response of cauliflower and broccoli plants to increased salinity which showed an increased dry matter content of the edible plant part. Therefore, it seems that wild *C. raphanina* spp. *mixta* were subjected to water stress conditions which increased dry matter content of leaves and therefore their nutrients content on a fresh weight basis. To the best of our knowledge this is the first report regarding the proximate analysis and the energetic value of the species which presents an interesting nutrition value. 

#### 3.1.2. Free Sugars Composition

Free sugars composition is presented in Table 1. The main detected sugars in both wild and cultivated plants of *C. raphanina* spp. *mixta* were trehalose (0.224 and 0.144 g/100 g fw, respectively), followed by fructose (0.170 and 0.166 g/100 g fw, respectively), sucrose (0.081 and 0.111 g/100 g fw, respectively) and glucose (0.073 and 0.092 g/100 g fw, respectively). Wild plants contained higher amounts of trehalose and total sugars than cultivated ones on a fresh weight basis, which is justified on the one hand by the concentration effect and on the other hand by the water stress conditions under which they were grown, since according to Petropoulos et al. [20] the increase of osmolytes such as sugars is a common tolerance mechanism of wild plants against stress. However, the increase of sugars content under stress conditions is not always the case, since the analysis of *Cichorium spinosum* plants grown under salinity stress showed a significant reduction which indicates that other compounds such as organic acids and tocopherols may play an osmoprotective and cell protective role [54,55]. However, when the calculation was carried out on a dry weight basis the cultivated plants showed a significantly higher content of individual and total sugars than wild plants. The higher sugar content in cultivated plants could have a significant effect on the taste of the leaves and therefore could affect consumer’s acceptance of the final product as already reported in other studies with wild plants cultivated under commercial conditions [9,18,54]. Differences in the developmental stage between wild and cultivated plants at harvest may also have a pivotal role in the observed differences since wild plants were harvested based on their phenology (marketable size) without having more information regarding the plant age and the number of days after the development of young leaves. These results are consistent with other reports, since according to Petropoulos et al. [19] and Poli et al. [56] sugars composition shows great variation with the stage of development. Considering that leaves of *Centaurea* species are well known and highly appreciated for their bitter taste due to their high content in sesquiterpene lactones such as germacranolides and eudesmanolides [12,27,57,58], taste should be determined through panel tests to evaluate the effect of increased sugars content and how they can affect consumers’ acceptance. To the best of our knowledge this is the first report regarding the free-sugars composition of the species and further agronomic factors that may affect individual sugars content and their possible effects on taste have to be tested. 

#### 3.1.3. Organic Acids Composition

Organic acids composition is presented in Table 2. Citric acid was the most abundant organic acid in both wild and cultivated plants, followed by malic acid in the case of wild plants and oxalic and malic acid in cultivated ones. Moreover, ascorbic and fumaric acid were detected in low amounts in wild plants, while in cultivated ones only traces of fumaric acid were present. Overall, cultivated plants had a higher content of total organic acids compared to wild ones on a fresh weight basis, indicating the possible induction of protective mechanisms against oxidative stress in cultivated plants [54,55]. In contrast to our study, Nemzer et al. [59] reported a higher total and individual organic acids content in cultivated purslane plants compared to wild ones, although different genotypes were tested in both cases which may partially justify this difference [60]. According to the literature, organic acids composition shows a great variability among wild edible species, depending on the edible part and the growing conditions [20,60,61,62,63]. Special focus is given on oxalic acid content which is undesirable when consumed in high amounts (daily consumption higher than 5 g for adults; [31]), since it reduces Ca availability and induces the formation of kidney stones [64,65]. The higher oxalic acid content observed in the cultivated plants compared to the wild ones could be explained by differences in pH level, nitrogen availability, and/or nitrogen form available in the growing medium and natural soil, respectively. Lower levels of oxalic acid have been observed in *Cichorium spinosum* L. [66], purslane [67] and spinach [68] with an increasing proportion of NH_4_-N versus NO_3_^-^N, which is likely to be observed in a natural soil versus a cultivated and more aerated soil. Yet, the high level of oxalic acid observed in cultivated plants could also be explained by a higher availability of total nitrogen in the cultivation system compared to the non-fertilized wild plants. These results are in agreement with the findings of Zhang et al. [68] who observed an increase of oxalic acid with increasing the amount of nitrogen available for the crop. Further research is warranted to investigate the effect of nitrogen rate and form on the nutritional profile of *C. raphanina* spp. *mixta*. Despite the higher oxalic acid content of cultivated *C. raphanina* spp. *mixta* plants observed in our study, the detected amounts are lower than those reported on a fresh weight basis in other wild (e.g., *Chenopodium album* [69], *Sonchus oleraceus* [61,70], *Amaranthus viridis* [71], *Silybum marianum* and *Beta maritima* [61], and purslane [60,62]) or cultivated species (e.g., spinach [72]) which are considered as rich sources of this anti-nutritional factor. Therefore, based on the set safe limit high amounts of *C. raphanina* spp. *mixta* leaves (more than 1182 g) have to be consumed to cause severe health effects. However, apart from oxalic acid content, Ca content is also important and Guil et al. [69] have suggested that the recommended safe ratio of oxalic acid/Ca has to be higher than 2.5. Therefore, apart from oxalic acid, also Ca content must be considered to make safe conclusions about the toxicity limits of wild edible greens. To the best of our knowledge this is the first report regarding the organic acids composition of the species, while special attention should be given to the high oxalic acid content in cultivated plants and agronomic practices that may help decreasing its content should be tested prior to suggesting its consumption by the wider public. 

#### 3.1.4. Tocopherols Composition 

The content of tocopherols is presented in Table 2. α- and γ-tocopherols were the only detected vitamin E vitamers, while cultivated plants contained higher amounts of both compounds than wild ones. In contrast, Fernández-Marín et al. [21] reported a variable response of different legumes to growing conditions (wild and domesticated) in terms of total tocopherols content in seeds with increasing or decreasing trends being depended on the species. Similarly to our study, α-tocopherol was reported to be the main tocopherol in various wild edible species which are regarded as rich sources of vitamin E contributing significantly to recommended daily allowance (RDA) [31,61,73,74,75], although β- and γ-tocopherol which were not detected in our study have been reported in other species [17,19,28,42,76]. Moreover, the higher amounts of tocopherols observed in cultivated plants compared to wild plants are probably associated with the activation of the antioxidant mechanism of plants for cell membrane protection, since cultivated plants were grown under completely different conditions (lower altitude, higher mean temperature) from wild ones and probably were subjected to stress [54,55]. Similarly to our study, Nemzer et al. [59] reported a higher α-tocopherol content in cultivated purslane plants compared to wild ones, suggesting that commercial cultivation is feasible for the production of nutritious edible greens. According to the literature, tocopherols content in plant tissues is correlated with the photosynthetic system functionality as they protect pigments and proteins involved in the photosynthesis process from oxidation [77,78], or they have non-antioxidant functions in membrane permeability and fluidity [79]. The higher amounts of total tocopherols also suggest that cultivated plants have a better nutritional value compared to wild ones, since the consumption of lower amounts of cultivated leaves compared to wild ones can cover a similar percentage of the RDA (15 mg/day for adults; [80]). More importantly, the higher α-tocopherol content indicates higher vitamin E bioavailability, since this tocopherol is preferably absorbed and distributed within human body [81]. To the best of our knowledge this is the first report regarding the tocopherols’ composition of the species.

#### 3.1.5. Fatty Acids Composition

Fatty acids composition is presented in Table 3. The most abundant fatty acids in both wild and cultivated plants were α-linolenic (38.2% and 29.5% in wild and cultivated plants, respectively), linoleic (25.5% and 28.2% in wild and cultivated plants, respectively) and palmitic acid (22.2% and 28.4% in wild and cultivated plants, respectively), while other fatty acids such as behenic, oleic and stearic acid were detected in lower amounts. The same fatty acids were also detected in other wild edible greens such as *Cichorium spinosum* [10,18,19,82], *Portulaca oleracea* [60,62,83] and other species [84,85]. Recently, Nemzer et al. [59] suggested significant differences in the fatty acids profile of wild and cultivated *Portulaca oleracea* plants, although the genotype was not the same in both cases to make safe conclusions regarding the effect of growing system. Moreover, in the study of Aktumsek et al. [86] the aerial parts from four *Centaurea* species (*C. pseudoscabiosa* subsp. *araratica*, *C. pseudoscabiosa* subsp. *araratica*, *C. salicifolia* subsp. *abbreviate* and *C. babylonica*) were analyzed for their fatty acids composition and although the major compounds were similar to all the species (linoleic, palmitic, linoleic and α-linolenic acid) significant differences in fatty acids profile were observed. Similarly, Erdogan et al. [87] and Tekeli et al. [88,89] studied the aerial parts of various endemic to Turkey *Centaurea* species and reported a significant variation in the fatty acids profile. Finally, apart from leaves the flowers of some *Centaurea* species are edible presenting a different fatty acids profile compared to leaves [90,91]. In our study, wild plants contained higher amounts of n-3 fatty acids (α-linolenic acid) than n-6 fatty acids (linoleic acid) compared to cultivated ones which were characterized by a balance between these two types of important dietary fats. This difference is also reflected in the classes of fatty acids with wild plants having a higher content of polyunsaturated fatty acids (PUFA), while cultivated plants were most abundant in monounsaturated and saturated fatty acids (MUFA and SFA, respectively). Furthermore, the ratios of PUFA/SFA and n6/n3 fatty acids are <4.0 and >0.45, respectively, in both wild and cultivated plants, although the values recorded for wild plants indicate a better nutritional value than cultivated ones in terms of fatty acids composition. 

#### 3.1.6. Phenolic Compounds Composition

Data on the identification of phenolic compounds are presented in Table 4 (retention time, λ_max_, pseudomolecular ion, major fragment ions in MS^2^, and the tentative identification of each individual compound). The corresponding quantification of each phenolic compound identified on the studied samples is presented in Table 4 as well. Twelve compounds were identified, 5 flavonols, 5 flavanones, and 2 flavones *O*-glycosylated. Although the information on the phenolic composition of *C. raphanina* spp. *mixta* is very scarce, the description of these compounds in other *Centaurea* species has been extensively studied by many authors [92,93]. However, the profile herein presented is very different from the other species, with the main difference being the absence of phenolic acids in these samples. Regarding the flavonols, peak 2 (quercetin-3-*O*-glucoside, [M-H]^−^ at *m/z* 477) was identified by comparing its retention time, mass spectra and UV-vis characteristics with the available commercial standard. A myricetin glycoside derivative was also found in the studied samples, presenting a pseudomolecular ion [M-H] ^−^ at *m/z* 493 and a unique MS^2^ fragment at *m/z* 317 (loss of 162 u), being tentatively identified as myricetin-*O*-glucoside. Finally, three kaempherol derivatives were tentatively identified, namely peaks 3 ([M-H]^−^ at *m/z* 461), 4 ([M-H]^−^ at *m/z* 579), and 7 ([M-H]^−^ at *m/z* 665), as kaempherol-*O*-glucuronide, kaempherol-*O*-hexosyl-pentoside, and kaempherol-*O*-malonyl-pentoside, respectively. The presence of myricetin, quercetin, apigenin, and kaempherol *O*-glycosylated derivatives was also reported in the water extracts of *C. raphanina* by Mikropoulou et al. [7]. Concerning the flavanone group, only one type of aglycone was found, namely pinocembrin (*m/z* at 255, and a typical UV-vis spectrum at 286 and shoulder at 328). This type of compound has been previously detected in *C. raphanina* water extracts by Mikropoulou et al. [7], where the presence of pinocembrin-*O*-arabirosyl-glucoside (peak 8, [M-H]^−^ at *m/z* 549), pinocembrin-*O*-neohesperidoside (peak 9, [M-H]^−^ at *m/z* 563), pinocembrin-*O*-acetylarabirosyl-glucoside (peak 10, [M-H]^−^ at *m/z* 591), and pinocembrin-*O*-acetylneohesperidoside isomer I and II (peaks 11 and 12 [M-H]^−^ at *m/z* 605, respectively) was also reported. The identification of the compounds detected in our study was performed based on the findings suggested by those authors. Finally, regarding the flavone group, peak 5, was tentatively identified as apigenin-*O*-hexosyl-pentoside, presented a pseudomolecular ion [M-H]^−^ at *m/z* 563 and a unique MS^2^ fragment at *m/z* 269 (loss of 162 u + 132 u, corresponding to the successive loss of one hexosyl and a pentosyl unit, respectively). Moreover, peak 6 ([M-H]^−^ at *m/z* 445), tentatively identified as apigenin-7-*O*-glucuronide is in accordance with the identification performed in *C. cyanus* L. dry petals by Pires et al. [93].

Regarding the quantification of phenolic compounds, the main detected compound was pinocembrin neohesperidoside (2.1 and 0.54 mg/g fw, in wild and cultivated plants, respectively), followed by pinocembrin acetyl neohesperidoside isomer I (0.62 and 0.08 mg/g fw, in wild and cultivated plants, respectively) and pinocembrin acetylarabirosyl glucoside (0.54 and 0.093 mg/g fw, in wild and cultivated plants, respectively). Wild plants contained higher amounts of all the detected compounds compared to cultivated plants, except for pinocembrin acetyl neohesperidoside isomer II which was detected in very low amounts in those plants. Similar results have been reported by Nemzer et al. [59] who also observed higher amounts of total phenolic compounds (TPC) in wild purslane plants compared to cultivated ones, although they also suggested that TPC content differed between the tested plant tissues (leaves, stems and whole plants). Moreover, Kim and Yoon [94] reported that wild plants of *Lactuca indica* L. contained higher amounts of polyphenols and flavonoids and presented a higher radical scavenging and anti-inflammatory ability than the cultivated ones. In contrast, Gutiérrez-Velázquez et al. [95] reported that cultivated plants of watercress (*Rorippa nasturtium* var. *aquaticum* L.) contained higher amounts of total phenolics than wild ones; however, the tested wild and cultivated genotypes were different to allow for a direct comparison, while total flavonoids and tannins prevailed in wild plants. The results of our study are very important for the determination of the growing system effect on phenolic compounds composition, since the same genotype was tested in both systems allowing for direct comparisons.

### 3.2. Bioactive Properties

#### 3.2.1. Antioxidant Activity

The antioxidant activity results determined through the OxHLIA and TBARS assays are presented in Table 5. The tested samples showed a different response depending on the assay with wild and cultivated plants showing better antioxidant properties for the TBARS and OxHLIA assays, respectively. These results indicate that different compounds are involved in the antioxidant mechanism in each case, e.g., phenolic compounds in wild plants and tocopherols in the case of cultivated ones. This finding is very common among natural matrices where different materials exhibit variable antioxidant activity depending on the applied assay and the extraction method, as well on the abundance of specific phytochemicals in each case [96,97,98]. For example, in contrast to our study Mikropoulou et al. [7] who studied *Centaurea raphanina* decoctions reported very low antioxidant activity in the DPPH assay compared to other wild edible greens probably due to the lack of hydroxyl groups in pinocembrin which was the most abundant phenolic compound detected in this species. However, in that study only the DPPH assay was tested which does not allow for safe conclusions regarding the antioxidant potential of the species. According to Morales et al. [76] tocopherols are associated with scavenging lipid peroxyl radicals thus inhibiting lipid peroxidation, which was also determined via the TBARS assay in our study. Similarly, Lockowandt et al. [42] reported a significant correlation of phenolic acids content in *C. cyanus* plant parts (edible flowers and non-edible parts) with antioxidant activity, although this class of phenolic compounds was not detected in our study. In the study of Lahneche et al. [99] *n*-butanol extracts of *C. sphaerocephala* which were richer in total phenolics and flavonoids exhibited higher antioxidant activity than other extracts. Moreover, several other reports tested the antioxidant activity of *Centaurea* species through various assays highlighting the potential role of different phytochemicals in such activities and the possible use of these species in human diet as antioxidant agents [86,100,101,102,103,104]. 

#### 3.2.2. Hepatotoxicity and Cytotoxic Activity

The results of cytotoxic effects of the extracts of wild and cultivated *Centaurea raphanina* spp. *mixta* leaves are presented in Table 6. A varied response was observed with cultivated plants showing better antiproliferative activity against breast carcinoma (MCF-7) and cervical carcinoma (HeLa) cell lines, while wild plants were more effective against hepatocellular carcinoma (HepG2) cell lines. Moreover, both the studied samples had mild toxicity against non-tumor (PLP2) and non-small lung cancer (NCI-H460) cell lines. The cytotoxic effects of the species have been recently studied by Mikropoulou et al. [7] who reported that water decoctions of leaves were effective against A5 metastatic spindle carcinoma cell lines and less effective against C5N immortalized keratinocyte cell lines. Moreover, Lockowandt et al. [42] who tested the antiproliferative activities of *C. cyanus* capitula reported no cytotoxicity against the same cell lines tested in our study. In another study, the aqueous extracts of *C. cyanus* petals showed low antiproliferative activity against HepG-2, Caco-2, and A549 cell lines [102], while Pires et al. [93] and Ostad et al. [105] reported no cytotoxicity for *C. cyanus* and *C. bruguierana* ssp. *Belangerana*, respectively. Therefore, it seems that although antiproliferative activities of *Centaurea* sp. against tumor and non-tumor cell lines depend on the species and the tested plant tissue [93,102,106], the extraction method and the used solvents are also important indicating that the detected activities are associated with specific compounds with different polarities and solvent-depended extraction efficiency [99,100,107]. To the best of our knowledge, this is the first report regarding the cytotoxic effects of *C. raphanina* spp. *mixta* against the studied cell lines and the results are promising for the commercial cultivation of the species in terms of its bioactive properties.

#### 3.2.3. Antimicrobial Properties

The antimicrobial properties of the extracts of wild and cultivated *Centaurea raphanina* spp. *mixta* leaves are presented in Table 7. The results showed higher antibacterial activity of wild plants than cultivated ones only against *Staphylococcus aureus* and *Bacillus cereus*, whereas no differences were observed for the rest of the tested bacteria. Moreover, the activity of the plant extracts was lower than the positive controls (streptomycin and ampicillin) for all the tested bacteria. Several studies have reported antibacterial activity for various *Centaurea* species in a species and pathogen depended manner [26], with sesquiterpene lactones being indicated as the main compounds responsible for these activities [12,108,109]. Similarly to our study, *Centaurea* species were reported as effective against *S. aureus*, *Escherichia coli* and *B. cereus* by Tekeli et al. [26], while Ćirić et al. [108] identified zuccarinin as the most potent sesquiterpene lactone isolated from another Greek endemic *Centaurea* species, namely *C. zuccariniana*. This class of sesquiterpenoids which are commonly found in species of the Asteraceae family exhibited strong antibacterial activity in many other species as for example *C. rhizantha* [110], *C. resupinata* subsp. *dufourii* [111], *C. pungens* [112], *C. ragusina* [113] and *C. behen* [114]. Pinocembrin and derivatives were the major phenolic compounds identified in our study and these could be associated with the antibacterial activity of the species since according to Micropoulou et al. [7] they possess significant bioactivities. 

The antifungal properties of plant extracts are presented in Table 7. The extracts from wild plants were more effective than cultivated plants against *Aspergillus versicolor*, *Trichoderma viride* and *Penicillium verrucosum* var. *cyclopium*, while their activity against *T. viride* was even higher than the used positive controls (bifonazole, ketoconazole). According to Panagouleas et al. [27] who studied the antifungal activities of the same species, from all the isolated compounds only cnicin showed significant effects against the tested fungi despite being detected in very low concentration (ca 0.0002%). They suggested that flavonoids are also responsible for the observed antifungal properties, with flavones being more potent than flavanones and flavonols [27]. This finding highlights the fact that natural matrices may have different response from isolated compounds and even minor components may show significant bioactivities. The antifungal properties of *Centaurea* species have been highlighted in several other reports. For example, Ćirić et al. [108] suggested that three newly found sesquiterpene lactones isolated from *C. zuccariniana*, namely one heliangolide and two eudesmanolides, possess significant antifungal activities against several fungi. Similarly, Koukoulitsa et al. [115] and Karioti et al. [116] found that sesquiterpene lactones were responsible for the observed antifungal activities of *C. deusta* and various endemic to Greece *Centaurea* species, respectively. These finding highlights the fact that natural matrices may have different response from isolated compounds and even minor components may show significant bioactivities. Moreover, apart from sesquiterpene lactone other secondary metabolites may contribute to the overall bioactive properties of these species, as already described by Micropoulou et al. [7].

## 4. Conclusions

Our results showed that commercial cultivation of *C. raphanina* spp. *mixta* is promising, since no adverse effects were observed on the nutritional value and the quality of the species. Most of the differences between wild and cultivated plants could be partly ascribed to the concentration effect due to lower moisture content of wild plants. In some cases (e.g., sugars, organic acids and tocopherols content, cytotoxicity) cultivated plants showed better results than wild plants, whereas a moderately high oxalic acid content was detected in cultivated plants without however exceeding the set limit for safe consumption. Nevertheless, from the consumer’s standpoint the findings of our study have great importance since the recommended daily intake of nutrients can be covered by the consumption of less amounts of wild plants, especially regarding phenolic compounds which were considerably higher than cultivated ones. On the other hand, the commercial cultivation of *C. raphanina* spp. *mixta* could make this product more easily available and affordable to consumers who could improve and diversify their diets with similar quality and without putting at risk agrobiodiversity from irrational harvesting of wild plants and the resulting genetic erosion. 

## Figures and Tables

**Table 1:** Nutritional value (g/100 g fw), energetic value (kcal/100 g fw) and composition in sugars (g/100 g fw) of wild and cultivated *Centaurea raphanina* spp. *mixta* leaves (mean ± SD).

Sample	Moisture	Fat	Protein	Ash	Carbohydrates	Energy
Wild	84.8 ± 0.3	0.42 ± 0.03	2.82 ± 0.01	2.17 ± 0.06	9.7 ± 0.1	54.1 ± 0.1
Cultivated	93.3 ± 0.4	0.189 ± 0.002	1.42 ± 0.04	1.08 ± 0.04	4.04 ± 0.01	23.5 ± 0.1
*t* test	<0.001	<0.001	<0.001	<0.001	<0.001	<0.001
**Sample**	**Fructose**	**Glucose**	**Sucrose**	**Trehalose**	**Total Sugars**	
Wild	0.170 ± 0.001	0.073 ± 0.001	0.081 ± 0.003	0.224 ± 0.005	0.550 ± 0.007	
Cultivated	0.166 ± 0.002	0.092 ± 0.002	0.111 ± 0.001	0.144 ± 0.004	0.510 ± 0.009	
*t* test	0.184	<0.001	<0.001	<0.001	<0.01	

Comparison of means of wild and cultivated plants of *Centaurea raphanina* spp. *mixta* was performed with Student’s *t*-test at *p* = 0.05.

**Table 2:** Composition in organic acids (mg/100 g fw) and tocopherols (mg/100 g fw) of wild and cultivated *Centaurea raphanina* spp. *mixta* leaves (mean ± SD).

Sample	Oxalic Acid	Malic Acid	Ascorbic Acid	Citric Acid	Fumaric Acid	Total Organic Acids
Wild	45.9 ± 0.1	387.1 ± 0.9	0.56 ± 0.06	409 ± 1	0.020 ± 0.001	842.8 ± 0.4
Cultivated	423 ± 1	320 ± 2	0.050 ± 0.001	460 ± 6	tr	1203 ± 6
*t-*test	<0.001	<0.001	<0.001	<0.001	-	<0.001
**Sample**	**α-Tocopherol**		**γ-Tocopherol**		**Total Tocopherols**	
Wild	0.045 ± 0.001		0.021 ± 0.001		0.070 ± 0.001	
Cultivated	0.185 ± 0.005		0.067 ± 0.002		0.260 ± 0.007	
*t-*test	<0.001		<0.001		<0.001	

tr—traces; comparison of means of wild and cultivated plants of *Centaurea raphanina* spp. *mixta* was performed with Student’s *t*-test at *p* = 0.05.

**Table 3:** Fatty acids composition (relative %) of wild and cultivated *Centaurea raphanina* spp. *mixta* leaves (mean ± SD).

Fatty Acids	Wild	Cultivated	*t*-Test
C8:0	0.56 ± 0.04	0.120 ± 0.006	<0.001
C10:0	0.048 ± 0.002	0.13 ± 0.01	<0.001
C11:0	0.13 ± 0.01	0.43 ± 0.01	<0.001
C12:0	0.387 ± 0.002	0.33 ± 0.02	<0.01
C14:0	1.12 ± 0.01	0.930 ± 0.008	<0.001
C14:1	0.094 ± 0.008	0.222 ± 0.001	<0.001
C15:0	0.54 ± 0.03	0.44 ± 0.01	<0.001
C16:0	22.2 ± 0.5	28.4 ± 0.4	<0.001
C17:0	0.28 ± 0.02	0.63 ± 0.03	<0.001
C18:0	2.42 ± 0.06	3.5 ± 0.2	<0.001
C18:1n9c	2.09 ± 0.01	2.94 ± 0.01	<0.001
C18:2n6c	25.5 ± 0.6	28.2 ± 0.1	<0.001
C18:3n3	38.2 ± 0.1	29.5 ± 0.1	<0.001
C20:0	0.382 ± 0.004	0.84 ± 0.04	<0.001
C21:0	0.49 ± 0.03	0.319 ± 0.008	<0.001
C22:0	4.1 ± 0.2	1.30 ± 0.03	<0.001
C23:0	0.48 ± 0.02	0.427 ± 0.004	<0.001
C24:0	1.04 ± 0.04	1.4 ± 0.2	<0.001
SFA	34.2 ± 0.6	39.16 ± 0.01	<0.001
MUFA	2.19 ± 0.02	3.17 ± 0.01	<0.001
PUFA	63.7 ± 0.6	57.67 ± 0.01	<0.001
PUFA/SFA	1.9 ± 0.3	1.47 ± 0.01	<0.001
n6/n3	0.67 ± 0.33	0.96 ± 0.12	<0.001

Caprylic acid (C8:0); capric acid (C10:0); undecylic acid (C11:0); lauric acid (C12:0); myristic acid (C14:0); pentadecylic acid (C15:0); palmitic acid (C16:0); margaric acid (C17:0); stearic acid (C18:0); oleic acid (C18:1n9); linoleic acid (C18:2n6c); α-linolenic acid (C18:3n3); arachidic acid (C20:0); heneicosylic acid (C21:0); behenic acid (C22:0); tricosylic acid (C23:0); lignoceric acid (C24:0); SFA: saturated fatty acids; MUFA: monounsaturated fatty acids; PUFA: polyunsaturated fatty acids; n6/n3: omega-6/omega-3 fatty acids. Comparison of means of wild and cultivated plants of *Centaurea raphanina* spp. *mixta* was performed with Student’s *t*-test at *p* = 0.05.

**Table 4:** Retention time (Rt), wavelengths of maximum absorption in the visible region (λ_max_), mass spectral data, tentative identification and quantification (mg/g of plant fw) of the phenolic compounds present in the extracts of wild and cultivated *Centaurea raphanina* spp. *mixta* leaves.

Peak	Rt (min)	λ_max_ (nm)	[M-H]^−^ (*m/z*)	MS^2^ (*m/z*)	Tentative Identification	Wild	Cultivated	*t*-Test
1	14.16	349	493	317 (100)	Myricetin-*O*-glucoside	0.099 ± 0.001	0.045 ± 0.001	<0.001
2	18.1	344	477	301 (100)	Quercetin-3-*O*-glucoside	0.036 ± 0.001	0.011 ± 0.001	<0.001
3	18.63	334	461	285 (100)	Kaempherol-*O*-glucuronide	0.113 ± 0.003	0.031 ± 0.002	<0.001
4	20.4	334	579	285 (100)	Kaempherol-*O-*hexosyl-pentoside	0.049 ± 0.001	0.016 ± 0.001	<0.001
5	22.14	334	563	269 (100)	Apigenin-*O*-hexosyl-pentoside	0.057 ± 0.001	0.02 ± 0.001	<0.001
6	22.9	334	445	269 (100)	Apigenin-*O*-glucuronide	0.043 ± 0.001	0.016 ± 0.001	<0.001
7	25.44	332	665	621 (100), 285 (45)	Kaempherol-*O*-malonyl-pentoside	0.034 ± 0.001	0.011 ± 0.001	<0.001
8	28.28	286/326	549	429 (12), 297 (14), 279 (5), 255 (41)	Pinocembrin-*O*-arabirosyl-glucoside	0.093 ± 0.001	0.023 ± 0.002	<0.001
9	29.47	286/326	563	443 (12), 401 (5), 297 (21), 255 (58)	Pinocembrin-*O*-neohesperidoside	2.1 ± 0.1	0.54 ± 0.01	<0.001
10	31.39	288/328	591	549 (30), 429 (20), 297 (15), 279 (5), 255 (32)	Pinocembrin-*O*-acetylarabirosyl-glucoside	0.54 ± 0.01	0.093 ± 0.001	<0.001
11	31.79	285/326	605	563 (12), 545 (5), 443 (30), 401 (10), 255 (40)	Pinocembrin-*O*-acetylneohesperidoside isomer I	0.62 ± 0.01	0.08 ± 0.01	<0.001
12	32.14	286/328	605	563 (10), 545 (5), 443 (28), 401 (9), 255 (39)	Pinocembrin-*O*-acetylneohesperidoside isomer II	6.68 ± 0.02	0.54 ± 0.03	<0.001
					Tfols	0.33 ± 0.01	0.113 ± 0.003	<0.001
					Tflav	10.1 ± 0.1	1.28 ± 0.04	<0.001
					Tflavone	0.100 ± 0.001	0.036 ± 0.001	<0.001
					TPC	10.5 ± 0.1	1.42 ± 0.04	<0.001

tr—traces; nd—not detected. Tfols: total flavonols; Tflav: total flavanones; Tflavone: total flavones; TPC: total phenolic compounds. Standard calibration curves used for quantification: apigenin-7-*O*-glucoside (*y* = 10683*x* − 45794, *R²* = 0.996, LOD = 0.10 µg/mL and LOQ = 0.53 µg/mL, peaks 5 and 6); myrcetin (*y* = 23287*x* − 581708, *R²* = 0.9988, LOD = 0.23 µg/mL and LOQ = 0.78 µg/mL, peak 1); naringenin (*y* = 18433*x* + 78903, *R²* = 0.9998, LOD = 0.17 µg/mL and LOQ = 0.81 µg/mL, peaks 8, 9, 10, 11, and 12); and quercetin-3-*O*-glucoside (*y* = 34843*x* − 160173, *R²* = 0.9998, LOD = 0.21 µg/mL and LOQ = 0.71 µg/mL, peaks 2, 3, 4, and 7). Comparison of means of wild and cultivated plants of *Centaurea raphanina* spp. *mixta* was performed with Student’s *t*-test at *p* = 0.05.

**Table 5:** Antioxidant activity of hydroethanolic extracts from wild and cultivated *Centaurea raphanina* spp. *mixta* leaves (mean ± SD).

Sample	OxHLIA (IC_50_; µg/mL); Δt = 60 min	TBARS (EC_50_, μg/mL)
Wild	35 ± 2	65 ± 2
Cultivated	83 ± 6	29 ± 1
Positive control Trolox	19.6 ± 0.1	23 ± 0.1
*t*-test	<0.001	<0.001

EC_50_: extract concentration corresponding to a 50% of antioxidant activity. Trolox EC_50_ values: 23 µg/mL (TBARS inhibition) and 19.6 µg/mL (OxHLIA Δt = 60 min). Comparison of means of wild and cultivated plants of *Centaurea raphanina* spp. *mixta* was performed with Student’s *t*-test at *p* = 0.05.

**Table 6:** Cytotoxicity and antitumor activity (GI_50_ values μg/mL) of hydroethanolic extracts from wild and cultivated *Centaurea raphanina* spp. *mixta* leaves.

Sample	Cytotoxicity to Non-Tumor Cell Lines	Cytotoxicity to Tumor Cell Lines			
	PLP2 (Porcine Liver Primary Culture)	HeLa (Cervical Carcinoma)	HepG2 (Hepatocellular Carcinoma)	MCF-7 (Breast Carcinoma)	NCI-H460 (Non-Small Cell Lung Cancer)
Wild	366 ± 22	322 ± 9	238 ± 14	>400	327 ± 21
Cultivated	369 ± 4	283 ± 24	>400	259 ± 2	314 ± 22
Positive control Ellipticine	2.3 ± 0.2	0.9 ± 0.1	1.10 ± 0.09	1.21 ± 0.02	1.03 ± 0.09
*t*-test	0.78	<0.001	<0.001	<0.001	0.34

GI_50_ values correspond to the sample concentration responsible for 50% inhibition of growth in tumor cells or in a primary culture of liver cells-PLP2. GI_50_ values for Ellipticine (positive control): 1.2 μg/mL (MCF-7), 1.0 μg/mL (NCI-H460), 0.91 μg/mL (HeLa), 1.1 μg/mL (HepG2) and 2.3 μg/mL (PLP2). Comparison of means of wild and cultivated plants of *Centaurea raphanina* spp. *mixta* was performed with Student’s *t*-test at *p* = 0.05.

**Table 7:** Antibacterial and antifungal activity (MIC, MBC, and MFC mg/mL) of hydroethanolic extracts from wild and cultivated *Centaurea raphanina* spp. *mixta* leaves and positive controls.

Sample	MIC/MBC	*S. aureus* (ATCC 11632)	*B. cereus* (Food Isolate)	*L. monocytogenes* (NCTC 7973)	*E. coli* (ATCC 25922)	*S. typhimurium* (ATCC 13311)	*E. cloacae* (ATCC 35030)
Wild	MIC *	0.5	0.5	1	0.5	2	2
	MBC	1	1	2	1	4	4
Cultivated	MIC	1	1	2	0.5	2	2
	MBC	2	2	4	1	4	4
Streptomycin	MIC	0.1	0.025	0.15	0.1	0.1	0.025
	MBC	0.2	0.05	0.3	0.2	0.2	0.05
Ampicillin	MIC	0.1	0.1	0.15	0.15	0.1	0.1
	MBC	0.15	0.15	0.3	0.2	0.2	0.15
**Sample**	**MIC/MFC**	***Aspergillus fumigatus*** **(ATCC 9197)**	***Aspergillus niger*** **(ATCC 6275)**	***Aspergillus versicolor*** **(ATCC 11730)**	***Penicillium funiculosum*** **(ATCC 36839**	***Trichoderma viride*** **(IAM 5061)**	***Penicillium verrucosum var. cyclopium*** **(food isolate)**
Wild	MIC	0.5	0.5	0.25	0.25	0.12	0.25
	MFC	1	1	0.25	0.5	0.25	0.5
Cultivated	MIC	0.5	0.5	0.5	0.25	0.25	0.5
	MFC	1	1	1	0.5	0.5	1
Bifonazole	MIC	0.15	0.15	0.1	0.2	0.15	0.1
	MFC	0.2	0.2	0.2	0.25	0.2	0.2
Ketoconazole	MIC	0.2	0.2	0.2	0.2	1	0.2
	MFC	0.5	0.5	0.5	0.5	1.5	0.3

* MIC = minimal inhibition concentration; MBC = minimal bactericidal concentration; MFC =minimal fungicidal concentration.
